# Effect of trimetazidine on incidence of major adverse cardiac events in coronary artery disease patients undergoing percutaneous coronary intervention: A protocol for systematic review and meta-analysis

**DOI:** 10.1097/MD.0000000000022918

**Published:** 2020-10-30

**Authors:** Kun Zhu, Yu-shui Zheng, Yong Fang

**Affiliations:** Department of Cardiology, Wanbei Coal and Electricity Group General Hospital, Suzhou, China.

**Keywords:** trimetazidine, major adverse cardiac event, coronary artery disease, percutaneous coronary intervention, protocol, systematic review

## Abstract

**Background:**

Percutaneous coronary intervention (PCI) is a common treatment method for coronary artery disease (CAD). PCI can cause myocardial ischemia or injury, and lead to major adverse cardiac events (MACEs). Trimetazidine has significant cardioprotective effects and improves endothelial dysfunction and myocardial injury. We will conduct a comprehensive systematic review and meta-analysis to evaluate effect of trimetazidine on incidence of MACE in CAD patients undergoing PCI.

**Methods:**

PubMed, Embase, Web of Science, Cochrane Library, the China National Knowledge Infrastructure, Chinese Biomedical Literature Database, and China Science and Technology Journal Database will be searched to collect randomized controlled trials (RCTs) of trimetazidine for CAD patients undergoing PCI. The range of publication time will be from the inception of the database to October 2020 without language limitation. Two reviewers will independently conduct study selection, data extraction and management, and assessment of risk of bias. Any disagreement will be resolved by discussion with the third reviewer. Review Manager Software 5.3 will be used for meta-analysis. The Cochrane risk of bias tool will be used to assess the risk of bias.

**Results:**

This study will provide a systematic synthesis of current published data to summarize the effect of trimetazidine on incidence of MACE such as stent restenosis, stent thrombosis, new significant coronary stenosis, myocardial infarction, heart failure, and cardiac arrest in CAD patients undergoing PCI.

**Conclusions:**

This meta-analysis will provide evidence as to whether trimetazidine can reduce incidence of MACE in CAD patients undergoing PCI.

**Study registration number:**

INPLASY202090083.

## 1 Introduction

With the rapid economic development, changes in lifestyles, and adoption of unhealthy diet, coronary artery disease (CAD) has become a major problem worldwide and the leading global cause of mortality in recent years.^[1–3]^ Percutaneous coronary intervention (PCI) is a primary and common treatment method for CAD.^[4]^ PCI can dilate stenotic or obstructed coronary artery, relieve clinical symptoms, and decrease mortality from CAD.^[5,6]^ However, PCI may induce coronary spasm, endothelial cell injury, and coronary artery distal embolization, which causes myocardial ischemia or injury.^[7–9]^ Major adverse cardiac events (MACEs) such as stent restenosis, stent thrombosis, new significant coronary stenosis, myocardial infarction, heart failure, and cardiac arrest are the main causes of mortality in CAD patients undergoing PCI.^[10,11]^ Obviously, it is of great significance to prevent the occurrence of MACE following PCI.

Trimetazidine is an anti-ischemic agent that exerts its cardioprotective effects by shifting cardiac energy metabolism from fatty acid oxidation to glucose oxidation, and reducing intracellular accumulation of hydrogen ions, lactate, sodium ions and calcium ions.^[12–14]^ One study showed that trimetazidine treatment could prevent the increase in creatine kinase-MB, cardiac troponin I, and von Willebrand factor and the decrease in nitric oxide induced by PCI, and improve endothelial dysfunction and myocardial injury.^[15]^ A meta-analysis showed that additional use of trimetazidine significantly improved the left ventricular ejection fraction and reduced elevated cardiac troponin I level and angina attacks during PCI.^[16]^

While up to now, no systematic review and meta-analysis has been performed on the effect of trimetazidine on incidence of MACE in CAD patients undergoing PCI. In view of this, we will conduct a comprehensive systematic review and meta-analysis to evaluate effect of trimetazidine for CAD patients undergoing PCI.

## 2 Methods

### 2.1 Study registration

This study has been registered on INPLASY (INPLASY202090083). This meta-analysis will be performed according the Preferred Reporting Items for Systematic Reviews and Meta-Analyses (PRISMA) statement checklist.^[17]^

### 2.2 Eligibility criteria for study selection

#### 2.2.1 Types of studies

All randomized controlled trials (RCTs) of trimetazidine for CAD patients undergoing PCI will be considered for inclusion without language limitation. Case reports, non-RCTs, animal experiments, reviews, and repeatedly published studies will be excluded.

#### 2.2.2 Types of participants

Participants who suffer from CAD and receive PCI treatment will be included without restrictions of the nationality, age, gender, and race.

#### 2.2.3 Types of interventions

In the treatment group, patients were given trimetazidine with no limitations of administration routes, dosage, or duration of intervention.

RCTs that have control groups with conventional treatments (such as drug therapy and physical therapy), or no treatment will be included.

#### 2.2.4 Types of outcomes

The incidence of MACE (such as stent restenosis, stent thrombosis, new significant coronary stenosis, myocardial infarction, heart failure, and cardiac arrest) will be designated as the outcomes.

### 2.3 Search strategy

PubMed, Embase, Web of Science, Cochrane Library, the China National Knowledge Infrastructure, Chinese Biomedical Literature Database, and China Science and Technology Journal Database will be searched to collect RCTs of trimetazidine for CAD patients undergoing PCI. The range of publication time will be from the inception of the database to October 2020 without language limitation. The detailed search strategy of PubMed is summarized in Table 1. The similar search strategies will be used for other electronic databases.

**Table 1:**
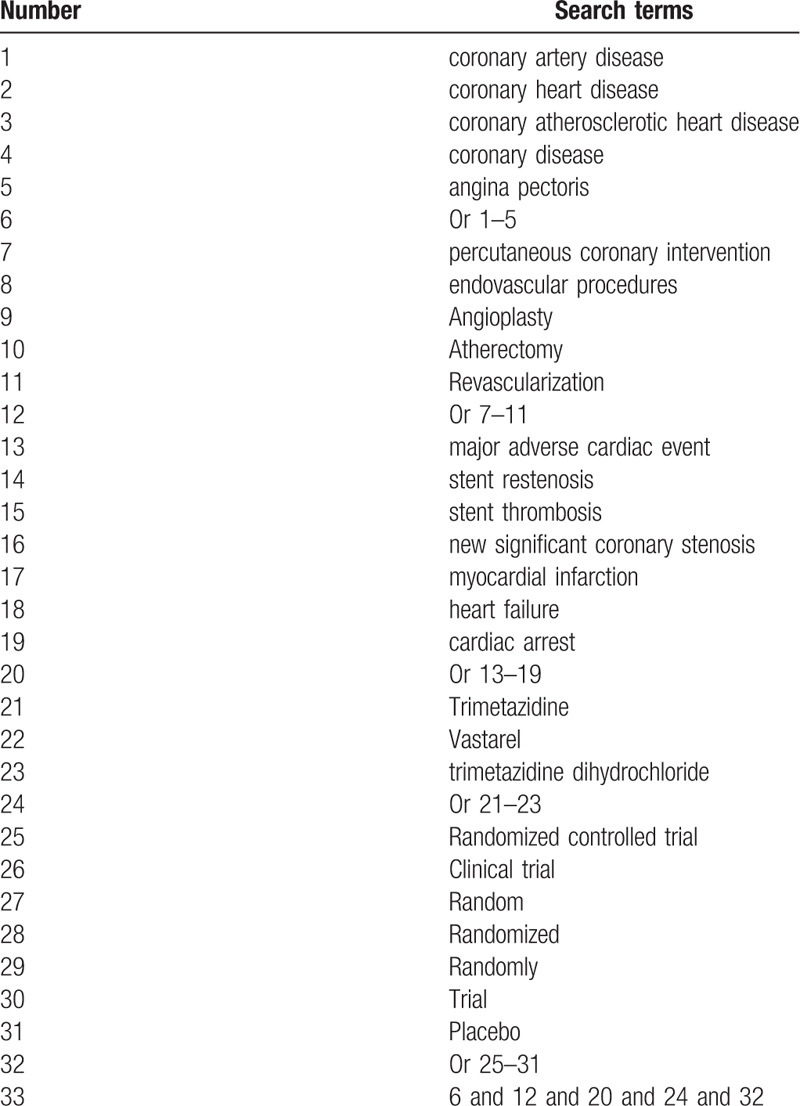
Search strategy of PubMed.

### 2.4 Selection of studies

All the searched articles will be exported to EndNote 7.0 (Thomas Reuters, CA) and duplicates will be excluded by software. Two reviewers will independently scan titles and abstracts to eliminate all irrelevant records. Then, the remaining records will be read by full texts in further assessing the inclusion of the study. Any disagreement about the selection of studies will be resolved by discussion with the third reviewer. A PRISMA flowchart will be designed to describe the details of selection process.

### 2.5 Data extraction and management

Data extraction will be conducted by 2 reviewers independently. A standard data extraction table will be designed according to Cochrane guidelines, including first author's name, country of publication, year of publication, title of journal, study design, patient information, intervention, control, duration of intervention, and specific details about MACE (number of persons experiencing stent restenosis, stent thrombosis, new significant coronary stenosis, myocardial infarction, heart failure, or cardiac arrest). Any disagreement about the data extraction will be resolved by discussion with the third reviewer. If some important information is missing, we will contact original authors by email to request detailed information about the research.

### 2.6 Assessment of risk of bias

Two reviewers will independently assess the risk of bias using the Cochrane risk of bias assessment tool. Seven items will be assessed, including random sequence generation, allocation concealment, blinding of participants and personnel, blinding of outcome assessment, incomplete outcome data, selective reporting, and other bias. A bias value of “high,” “unclear,” or “low” was evaluated for each item. Any disagreement about assessment of risk of bias will be resolved by discussion with the third reviewer.

### 2.7 Data synthesis and analysis

#### 2.7.1 Data synthesis

Review Manager Software 5.3 will be used for data synthesis. Risk ratio will be used for dichotomous outcomes with 95% confidence interval. The random effects model or fixed effects model will be selected according to the *I*^2^ value. Heterogeneity will be examined using the *I*^2^ test. The *I*^2^ value > 50% means significant heterogeneity, and the random effects model will be used. Otherwise, the *I*^2^ value ≤ 50% means minor heterogeneity, and the fixed effects model will be utilized. If significant heterogeneity still exists after subgroup analysis, meta-analysis will not be pooled, and descriptive summary will be reported.

#### 2.7.2 Subgroup analysis

Subgroup analysis will be performed to check the potential heterogeneity and inconsistency based on the different participant characteristics, administration routes and dose of trimetazidine, control methods, and outcome indicators.

#### 2.7.3 Sensitivity analysis

Sensitivity analysis will be applied to check the robustness and reliability of pooled results. We will perform meta-analysis again after eliminating studies in low quality and will apply different statistical methods.

#### 2.7.4 Reporting bias

Publication bias will be assessed with funnel plot and Egger regression test if sufficient trials (≥10 trials) are included.^[18,19]^

### 2.8 Ethics and dissemination

Ethical approval is not necessary because this study is based on literature analysis. The results of this study will be published in a peer-reviewed journal.

## 3 Discussion

To our knowledge, this is the first systematic review and meta-analysis to conduct a comprehensive literature search and provide a systematic synthesis of current published data to summarize the effect of trimetazidine on incidence of MACE in CAD patients undergoing PCI. We will search 7 electronic literature databases to avoid missing any potential eligible studies, and apply rigorous methodology to examine studies reporting the MACE of trimetazidine for CAD patients undergoing PCI. We believe that this systematic review and meta-analysis will provide clinical evidence for the trimetazidine treatment of CAD patients undergoing PCI, inform our understanding of the value of trimetazidine in treating CAD and reducing incidence of MACE following PCI, and provide helpful evidence for clinical practice, patients, and future research.

## Author contributions

**Conceptualization:** Kun Zhu.

**Data curation:** Kun Zhu, Yu-shui Zheng.

**Formal analysis:** Kun Zhu, Yu-shui Zheng, Yong Fang.

**Methodology:** Yu-shui Zheng, Yong Fang.

**Project administration:** Kun Zhu.

**Resources:** Kun Zhu, Yong Fang.

**Software:** Yu-shui Zheng, Yong Fang.

**Supervision:** Kun Zhu.

**Writing – original draft:** Kun Zhu, Yu-shui Zheng.

**Writing – review & editing:** Kun Zhu, Yu-shui Zheng, Yong Fang.
